# Clinical excellence: evidence on the assessment of senior doctors' applications to the UK Advisory Committee on Clinical Excellence Awards. Analysis of complete national data set

**DOI:** 10.1136/bmjopen-2016-011958

**Published:** 2016-06-02

**Authors:** John L Campbell, Gary Abel

**Affiliations:** 1 University of Exeter Medical School, Exeter, UK; 2 Primary Care Unit, University of Cambridge, Cambridge, UK

**Keywords:** reliability, clinical excellence, quality

## Abstract

**Objectives:**

To inform the rational deployment of assessor resource in the evaluation of applications to the UK Advisory Committee on Clinical Excellence Awards (ACCEA).

**Setting:**

ACCEA are responsible for a scheme to financially reward senior doctors in England and Wales who are assessed to be working over and above the standard expected of their role.

**Participants:**

Anonymised applications of consultants and senior academic GPs for awards were considered by members of 14 regional subcommittees and 2 national assessing committees during the 2014–2015 round of applications.

**Design:**

It involved secondary analysis of complete anonymised national data set.

**Primary and secondary outcome measures:**

We analysed scores for each of 1916 applications for a clinical excellence award across 4 levels of award. Scores were provided by members of 16 subcommittees. We assessed the reliability of assessments and described the variance in the assessment of scores.

**Results:**

Members of regional subcommittees assessed 1529 new applications and 387 renewal applications. Average scores increased with the level of application being made. On average, applications were assessed by 9.5 assessors. The highest contributions to the variance in individual assessors' assessments of applications were attributable to assessors or to residual variance. The applicant accounted for around a quarter of the variance in scores for new bronze applications, with this proportion decreasing for higher award levels. Reliability in excess of 0.7 can be attained where 4 assessors score bronze applications, with twice as many assessors being required for higher levels of application.

**Conclusions:**

Assessment processes pertaining in the competitive allocation of public funds need to be credible and efficient. The present arrangements for assessing and scoring applications are defensible, depending on the level of reliability judged to be required in the assessment process. Some relatively minor reconfiguration in approaches to scoring might usefully be considered in future rounds of assessment.

**Strengths and limitations of this study**

- First comprehensive published analysis of the reliability of the assessment processes used to evaluate applications from UK senior doctors for clinical excellence awards.
- Used comprehensive national data drawn from all of the 16 subcommittees assessing applications in the UK from the most recent completed round of applications (2014–2015).
- Quantified variances from a number of sources contributing to the assessments.
- Assumed the assessor variance to be constant across different types of assessor.
- Assumed that the assessor variances are constant across committees.

## Background

The UK Clinical Excellence Awards Scheme recognises and rewards National Health Service (NHS) consultants and senior academic GPs who perform over and above the standard expected of their role. It is managed and overseen by the Advisory Committee on Clinical Excellence Awards (ACCEA).^1^ The scheme recognises 12 levels of award—from local awards considered and awarded using a local process, through to nationally assessed and awarded bronze, silver, gold and platinum awards. Awards are associated with an increasing scale of monetary payments. New applications are assessed on the basis of individuals providing evidence of performance demonstrating sustained commitment to patient care and public health; high standards of clinical care, and commitment to the values and goals of the NHS.

Assessors consider applications guided by the principles of equity, transparency and value for money. ACCEA is a UK non-departmental public body. The Committee advises government ministers on award nominations. Applications for national level awards are assessed by the members of 1 of 14 regionally distributed subcommittees and from 2 national committees. Subcommittee membership is drawn from a wide range of professional, lay and NHS management (employer) backgrounds. Subcommittee members assess applications, identifying a shortlist of ranked nominees, which are then submitted for consideration to the main national ACCEA committee. In making their recommendations to ministers, ACCEA also considers doctors who have been shortlisted by a range of accredited national nominating bodies. Each application covers five domains of professional activity (service delivery, development, leadership, research and innovation, training), an outline of the doctor's job plan and a personal statement submitted by the doctor. Applications are submitted using an online process, which are assessed against strictly defined criteria (table 1) using a four-point scoring system in which each of the five domains of the application is awarded a score of 0, 2, 6 or 10 points. An overall average score is derived for each domain for each applicant and is used to rank applicants on a percentile basis.

**Table 1:** Domains and criteria adopted in the assessment of ACCEA applications

Domain 1—delivering a high quality service	Evidence should show achievements in delivering a service which is safe, has measurably effective clinical outcomes, provides good patient experience and where opportunities for improvement are consistently sought and implemented.
0 (Does not meet contractual requirements or when insufficient information has been produced to make a judgment.)	
2 (Meets contractual requirements)	Performance in some aspects of the role could be assessed as ‘over and above’ expected standards. But generally, on the evidence provided, contractual obligations are fulfilled to competent standards and no more.
6 (Over and above contractual requirements)	Some duties are performed in line with the criteria for ‘Excellent’, as below. However, on the evidence provided, most are delivered above contractual requirements, without being in the highest category.
10 (Excellent)	Applicants could show evidence of performance over and above the standard expected in one or more of the following (this list is not exhaustive): Contracted job is carried out to the highest standards. Evidence for this should come from benchmarking exercises or objective reviews by outside agencies. Where this is not available, there should be other evidence that the work undertaken is outstanding—in relation to service delivery and outcomes—when compared to that of peers. Personal role in service delivery by a team, with evidence of outstanding contribution, such as awards, audits or publications. Exemplary standards in dealing with patients, relatives and all grades of medical and other staff. Applicants should ideally include reference to a validated patient or carers’ survey or feedback on the service (external or peer review reports).
Domain 2—developing a high quality service	Evidence should show how applicants have significantly enhanced clinical effectiveness (the quality, safety and cost-effectiveness) of services locally and more widely within the NHS if this is the case.
0 (Does not meet contractual requirements or when insufficient information has been produced to make a judgement)	
2 (Meets contractual requirements)	The applicant has fully achieved their service-based goals and provided comprehensive services to a consistently high level. But there is no evidence of them making any major enhancements or improvements.
6 (Over and above contractual requirements)	The applicant has made high quality service developments, improvements or innovations that have contributed to a better and more effective service delivery. This could be demonstrated by: Improvement in service based on evidence. Improved outcomes (clinical effectiveness). Greater cost-effectiveness. Services becoming more patient centred and accessible. Benefits in prevention, diagnosis, treatment or models of care.
	For this score, the activity would be expected at local and possibly regional level—especially if in the face of difficult circumstances or constraints.
10 (Excellent)	In addition to some or all of the achievements listed in 6, applicants could show evidence of performance over and above the standard expected in one or more of the following (this is not exhaustive): Service innovation—introduction of new procedures, treatments or service delivery, based on original research or development or effectively overcoming barriers to clinical effectiveness. This should be backed up by relevant, completed audit cycles or research that has been adopted at regional, national or international level, with demonstrable change in evidence based practice. Clinical governance—introduction or development of clinical governance approaches, which have resulted in audited/published advances taken up elsewhere. Leadership in the development of the applicant's specialty at regional, national or international level. This should include evidence of wide participation in promoting the development of evidence-based practice in the specialty, including patient and public involvement.
Domain 3—leadership and managing a high quality service	Evidence should show how applicants have made a substantial personal contribution to leading and managing a local service or national/international service or health policy development.
0 (Does not meet contractual requirements or when insufficient information has been produced to make a judgement)	
2 (Meets contractual requirements)	Applicants should receive this score if they provide evidence of successfully contributing to the running of a trust or unit, especially in difficult circumstances, and maintaining excellent staff relations—by encouraging colleagues in nursing and other professionals ancillary to medicine.
6 (Over and above contractual requirements)	To score 6 points, applicants must show successful management skills, especially in innovative development and hard pressed services. They may also have been involved in recognised advisory committee work, at area and particularly national level (especially if as secretary or chair). Other criteria that would merit this score include effective chairing of a trust or university committee as, for example, clinical director. Look also for examples of how applicants have carried out appraisals for peers/non-career grade doctors or been involved in major reviews, enquiries or investigations or as part of a College/Specialty Advisory Committee. ACCEA does not expect to reward membership of such committees in itself. You should look for evidence that the contribution made by the applicant has been over and above expectations.
10 (Excellent)	In addition to some achievement acquiring a score of 6, applicants scoring 10 in this domain will have shown evidence of outstanding administrative achievement in a leadership role—as confirmed by their employer and/or other citations. Medical directors and other clinical managers should not be given this score purely because they hold the post—there must be clear evidence that they have distinguished themselves by leadership in advancement of health policy and delivery.
	Other evidence that could merit this score includes (this list is not exhaustive): Involvement in shaping national policy, aimed at modernising health services (might include effective chairing of an area or national importance advisory committee). Successful directorship of a large nationally recognised unit, institute or supraregional services. Planning and delivery of area or nationwide services. Other evidence from citations of exceptional activity and achievement.
Domain 4—research and innovation	Evidence should show how applicants have made a contribution to research or the evidence/evaluative base for quality or service innovation including the translation of evidence in to practice.
	Assessors should note evidence of the impact of research on improvement in healthcare and health.
0 (Does not meet contractual requirements or when insufficient information has been produced to make a judgement)	
2 (Meets contractual requirements)	If the applicant is an academic consultant, they should be considered by their employer to be ‘research active’—at a level commensurate with their contract. This rating would be based on the applicant's research output and associated publications within the past 5 years.
	If he or she is an NHS consultant, they will have undertaken clinical research, alone or in collaboration, which has resulted in publications. Or they may have collaborated actively in basic research projects established by others. They may also have actively encouraged research by junior staff and supervised their work.
6 (Over and above contractual requirements)	There will be evidence of the applicant having made a sustained personal contribution in basic or clinical research which could be demonstrated by: A lead or collaborative role, holding, or having held within the past 5 years, peer reviewed grants. A role as a major collaborator in clinical trials or other types of research. A publication record in peer-reviewed journals within the past 5 years. Supervision now, or in the past 5 years, of doctorate/post-doctorate fellows. Other markers of research standing such as lectures/invited demonstrations. Development of a method, a tool or equipment, which contribute to the understanding of, or towards care delivery.
10 (Excellent)	In addition to some or all of the achievements listed in 6, applicants could show evidence of performance over and above the standard expected in one or more of the following (this list is not exhaustive): Major peer-reviewed grants held currently and/or within the last 5 years, for which the applicant is the principal investigator or main research lead. They should have included the title, duration and value. Contribution to research and the evidence/evaluative base for quality. Research publications in high citation journals. National or international presentations/lectures/demonstrations given on research. Supervision of successful doctorate students, some of whom might have come on national or international fellowships. Patent of a significant innovation. Other peer determined markers of research eminence.
Domain 5—teaching and training	Evidence should show how teaching and training forms a major part of the contribution applicants make to the NHS, over and above contractual obligations.
0 (Does not meet contractual requirements or when insufficient information has been produced to make a judgement)	
2 (Meets contractual requirements)	Evidence of having fulfilled the teaching/training expectations identified in the job plan, in terms of quality and quantity.
6 (Over and above contractual requirements)	Applicants could present evidence in the following areas:
	The quality of teaching and/or training through regular audit and mechanisms such as 360° appraisal. This should include evidence of adaptation and modification, where appropriate, of these skills as a result of this feedback. Involvement in quality assurance of teaching and evidence of success with regulatory bodies involved with teaching and training. High performance in formal roles such as working with under and postgraduate deans, and involvement with postgraduate educational programmes in roles such as head of training/programme director, regional adviser and clinical tutor.
10 (Excellent)	In addition to some or all of the achievements listed in 6, applicants could show evidence of performance over and above the standard expected in one or more of the following (this list is not exhaustive): Leadership and innovation in teaching, including new course development innovative assessment method introduction of new learning techniques authorship of successful textbooks or other media on teaching/training. National and international educational leadership, such as presentations, invitations to lecture, peer reviewed and other publications on educational matters. Innovation and trend setting in teaching and training, including examination processes, for a college, faculty, specialist society or other national professional bodies.

ACCEA, Advisory Committee on Clinical Excellence Awards.

Various safeguards are built in to the assessment process with a view to ensuring a rigorous, fair and scientifically defensible outcome. Applications may be supported by a short citation submitted as part of the application from supporting individuals or from a number of recognised nominating bodies and authorities. Members of each subcommittee are divided into two approximately equally sized groups. One half score the bronze applications and the other half score the other levels. In general, a member will score all applications for a given level. Some individuals may occasionally elect not to score a particular application, for example, when perceived personal conflict of interest exists. In those circumstances, the mean of the remaining assessors' scores is applied to the ‘missing’ assessment. Following secure online submission of scores, subcommittees meet on two occasions to consider their scores and the ranking of applicants, considered in the light of an indicative number of awards suggested to the subcommittee for each level of award by the main national committee in advance of the subcommittee meeting. This indicative number is used to determine a regional cut-off score against which renewal applications from the same level are judged. If any uncertainty remains, individual applicants may be referred for further consideration and re-scoring by a national rescoring (NRES) committee. Platinum applicants are initially scored within the subcommittee process, but all are also secondarily and independently scored by a ‘second-level’ committee constituted at the national level to review and independently score all applications for this, the highest level of award. Following consideration at subcommittee level, a ranked list of recommendations is made to the central ACCEA committee for further review and final recommendation for funding to the responsible government minister.

### Assessment validity

The validity of assessment is complex, conceptually and in its evaluation. Downing^2^ ^3^ summarises validity under five headings (content, response process, internal structure, relationship with other variables and consequences), noting that each component is interlinked and should not be seen in isolation. In respect of the ACCEA application process, the domains being assessed and the criteria adopted for scoring are explicitly defined in published national guidance for assessors^4^ and made available within each application (table 1).

The analysis undertaken in this study explores the internal structure of the assessment process and specifically its reliability. This is a fundamental component of an overall evaluation. Derived reliability coefficients describe the percentage of variability that can be attributed to true differences between applicants. A coefficient of 0.8 implies that 80% of the variance in scores awarded to applicants is attributable to true differences between the applicants themselves, representing the ‘signal’ that the assessment system is seeking to detect (the remaining 20% being error—‘noise’—such as might be attributable to assessor variability for example). By using variance estimates, modelling can be undertaken to calculate what would happen to the reliability of the assessment if, for example, applications were scored by fewer or greater numbers of assessors.^5^ ^6^

In a time of financial austerity and of increased scrutiny of spending of public finances, there is a great need for evident transparency and streamlining in respect of resource allocation assessment and distribution. The aim of this study was to examine the statistical reliability of the subcommittee assessment process for new applications received during the 2014 award round and to provide guidance for the UK ACCEA committee on the rational use of assessment resources.

## Materials and Methods

Data were made available on a fully anonymised basis by the UK Department of Health relating to applications for national level clinical excellence awards considered by members of subcommittees plus the scores of the national committee assessing new platinum level applications. Data related to assessments made in the 2014 application round; awards from these applications were made in 2015. Analyses were undertaken in Stata V.13.1 (StataCorp College Station, Texas, USA). All data were complete apart from the assessor background role for the national committee assessing new platinum level applications. The mean score awarded to each applicant by each assessor was used for all analyses.

ACCEA also administers a parallel, historical scheme of ‘A’ (gold equivalent) and ‘B’ (bronze equivalent) award renewal applications whose introduction predated the present arrangements, which were introduced in 2003. Renewals of historical ‘A’ and ‘B’ awards were excluded from analysis based on four preliminary observations: (i) these were all renewal applications, (ii) the basis on which the award was made was historical and may have been different from the criteria pertaining now, (iii) initial analysis showed that ‘A’ renewals were, on average, much lower scoring than the equivalent gold renewals and (iv) including these applications (16 in total) would have potential to affect our analysis in an unpredictable way.

We described the distribution of applications by subcommittee, between subcommittees and across new and renewal applications. Since scores had been given on the basis of the award level applied for (ie, the same application assessed for a bronze award would likely score higher than if assessed for a gold award), all analyses were stratified by award level. We also further stratified our analysis by new applications versus renewals, as we would a priori expect the variance of renewals to be smaller (these applicants previously been judged to meet the minimum standard and so are likely to be more similar to each other than new applicants). We quantified the mean number of assessments made per applicant, by subcommittee and by award level. For comparison, we also described the number of subcommittee members submitting assessments within each committee. The two ‘national’ committees, assessing applicants from the Department of Health and the NRES committee, were considered to be regional subcommittees for analysis purposes. Box plots were used to illustrate the distribution of scores for each award level separately for new applications and renewals.

A series of multilevel random intercepts models were used to estimate the variance components attributable to four different sources; the subcommittee 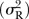, the assessors 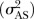, the applicants 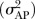 and residual variance 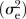. As applicants received scores from multiple assessors and assessors score multiple applicants, neither can be considered to be nested within the other and rather we consider them to be ‘crossed’.^7^ Applicants and assessors are nested within subcommittee, and the residual variance is nested within applicants and assessors. The subcommittee variance may either reflect systematic variation in the scores given by different subcommittees (conditional on the assessors making up those subcommittees) or systematic differences in the mean score of applicants (ie, the quality of applicants is higher in some subcommittees than others). Since no assessors and no applicants sit within more than one subcommittee, it is impossible to distinguish the relative contribution of these two components, with the exception of the new platinum applications, which are discussed below. The assessor variance captures systematic differences in the scores given by different assessors when they have assessed the same candidates. The applicant variance captures the true variance between applicants and can be considered the signal the system is aiming to capture. The residual variance can be considered noise and can be thought of as capturing the differences in scores that occur when assessors, who on average score the same, will give different scores to any one candidate. It also captures the possibility that the same assessor may give a different score to the same candidate at different times.

With the platinum awards, we compare two models, both including a random effect for subcommittee, but one based on the subcommittee scores and one based on the national committee scores. In the later model, the subcommittee variance relates to the committee applied to, that is, the region of the candidate rather than the different committees themselves. Comparing these two models gives insight into the source of the committee level variance.

Since judgement about awards are made by subcommittees, which apply specific regional cut-offs to decide when awards are recommended (rather than applying national cut-offs), we estimated reliabilities on the basis of scores derived within subcommittee rather than as a part of a national spectrum. In this situation, where all applicants at a given level are assessed by all of the same assessors, any variation in the hawkish/dovish tendency of individual assessors will not affect reliability, as the same exaggeration/suppression of scores will apply equally to all candidates within a subcommittee. Thus, the assessor variance does not influence reliability (λ) which is given by:

1
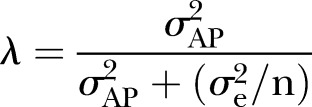
where n is the number of assessments made per candidate.

We also estimated the reliability where the make-up of groups of assessors is not consistent for candidates being judged against one another, for example as would be the case if national scoring was used, or not all assessors within a committee saw all applications being assessed (see online supplementary material). In this situation, differences between assessors become important and so we estimate differences in scores given by assessors of different background roles and its impact on reliability (see online supplementary material).

10.1136/bmjopen-2016-011958.supp1 Supplementary data

Finally, since the applicants scored by the NRES committee have already been scored by regional committees and were considered to be borderline cases, we also undertook a sensitivity analysis to investigate the contribution of the NRES committee scores to the overall results. We repeated the models used to estimate the variance components having excluded the NRES committee scores.

## Results

In 2014, 1916 applications were scored by members of 16 subcommittees (identified as A-P hereafter). Table 2 shows how these applications were shared between new applications (1529) and renewals (387) and across the various awards and subcommittees. New bronze award applications were the most numerous (650), closely followed by new silver applications (614). The smallest group was gold renewals with only 11 applications. Each subcommittee scored between 45 and 225 applications.

**Table 2:** Number of applications by subcommittee, level and application type

Regional subcommittee	New applications					Renewals					Overall total
	Bronze	Silver	Gold	Platinum	Total	Bronze	Silver	Gold	Platinum	Total	
A	17	17	6	0	40	9	6	0	1	16	56
B	16	15	3	2	36	7	2	0	0	9	45
C	40	43	9	3	95	28	5	1	2	36	131
D	36	34	12	4	86	9	6	0	1	16	102
E	80	73	27	8	188	26	8	1	2	37	225
F	26	38	13	7	84	12	4	2	2	20	104
G	48	40	14	4	106	27	8	2	5	42	148
H*	33	31	9	0	73	7	1	0	0	8	81
I	29	37	7	2	75	13	5	1	0	19	94
J	37	39	14	2	92	12	6	1	2	21	113
K	77	48	8	4	137	18	7	2	3	30	167
L	17	19	5	0	41	18	2	1	0	21	62
M	50	50	15	1	116	23	6	0	2	31	147
N	49	27	7	0	83	15	2	0	0	17	100
O	39	52	10	3	104	20	7	0	2	29	133
P	56	58	15	2	131	26	7	0	2	35	166
Total	650	621	174	84	1529	270	82	11	24	387	1916

*National rescoring committee (NRES).

**Figure 1:**
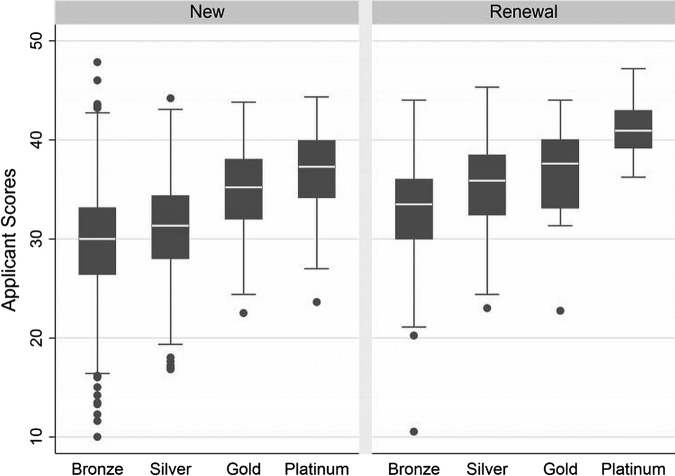
Box plot showing the variation in applicant scores by level and application type.

Figure 1 shows the distribution of scores for new applications and renewals, stratified by award level, showing that the mean score increases with increasing level, as expected. Further, the renewal scores tend to be higher than the new scores of the corresponding level. Figure 1 also illustrates the decreasing range of scores with increasing award level, and smaller range in renewal applications than that in new applications.

Overall, each application is marked by a mean of 9.5 assessors. Table 3 shows the mean number of assessors marking each application at each level within each committee. Typically, the number is between 9.3 and 10.5, although it does reach as low as 5 for some awards in one committee. Comparing table 3 with table 4, which shows the number of assessors making up each committee, shows that the vast majority of applications were scored by all committee members. The highest number of assessors used was 12.

**Table 3:** Mean number of assessors per application by subcommittee, level and application type

Regional subcommittee	New applications				Renewals			
	Bronze	Silver	Gold	Platinum	Bronze	Silver	Gold	Platinum
A	10.00	12.00	12.00	NA	9.00	12.00	NA	12.00
B	6.00	6.00	5.00	6.00	6.00	6.00	NA	NA
C	10.00	8.00	8.00	8.00	9.89	8.00	8.00	8.00
D	8.00	10.00	10.00	8.50	8.00	10.00	NA	10.00
E	11.00	10.96	10.85	10.00	10.00	11.00	11.00	11.00
F	9.00	11.00	11.00	10.00	9.00	11.00	11.00	11.00
G	8.96	8.98	9.00	8.00	8.96	9.00	9.00	9.00
H*	10.00	10.00	9.89	NA	5.00	5.00	NA	NA
I	12.00	12.00	12.00	9.50	11.00	12.00	12.00	NA
J	10.00	10.00	10.00	10.00	10.00	10.00	10.00	10.00
K	10.00	9.98	9.00	9.00	9.00	9.00	10.00	10.00
L	11.00	11.00	10.80	NA	11.00	10.50	10.00	NA
M	8.90	8.96	8.67	8.00	8.91	9.17	NA	8.50
N	10.00	7.00	7.00	NA	10.00	7.00	NA	NA
O	9.97	10.00	10.00	8.00	8.00	10.00	NA	10.00
P	10.00	8.98	10.00	10.00	10.00	10.00	NA	10.00
Total	9.83	9.77	9.82	10.50	9.31	9.83	10.09	9.75

*National rescoring committee (NRES).

NA, not available.

**Table 4:** Number of assessors on each subcommittee committee

Regional subcommittee	Bronze	Silver/gold/ platinum	Total
A	10	12	22
B	6	6	12
C	10	8	18
D	8	10	18
E	11	11	22
F	9	11	20
G	9	9	18
H*	10	10	20
I	12	12	24
J	10	10	20
K	10	10	20
L	11	11	22
M	9	10	19
N	10	7	17
O	10	10	20
P	10	10	20

*National rescoring committee (NRES).

Table 5 shows the estimated variance components of individual assessor scores of applications attributable to subcommittee, assessors, participants and residual variation for each of the award levels and application types. In each case, the highest variances are either that attributable to assessors or the residual variance, highlighting the need for the use of multiple assessors for each application. In nearly all cases, the estimated levels of variance attributable to these two sources are broadly similar. The exception is for the gold renewals where the estimates need to be treated with caution due to the very low sample size. The percentage of total variance attributable to the applicant decreases from 28% in new bronze applications to 11% in new platinum applications scored by the subcommittees. The amount of variance attributable to the subcommittee is always small (≤4% in all except gold renewals), and the fact that it is present in platinum new applications when scored by subcommittees but absent when scored by the national committee suggests that this variance is attributable to different subcommittees scoring differently rather than the standard of candidates varying between subcommittees. However, the size of this variance is small and the uncertainty is large, and so this interpretation must be treated with some caution.

**Table 5:** Variance components in the ACCEA assessment process by level and application type

	New applications					Renewals				
	Variance in scores (% of total variance) attributable to									
	Subcommittee	Assessors	Applicant	Residual	Total	Subcommittee	Assessors	Applicant	Residual	Total
Bronze	1.59 (1.8%)	30.05 (34.5%)	24.28 (27.8%)	31.28 (35.9%)	87.20 (100.0%)	1.74 (2.4%)	30.43 (41.5%)	14.02 (19.1%)	27.17 (37.0%)	73.36 (100.0%)
Silver	2.12 (2.7%)	32.20 (40.8%)	14.31 (18.1%)	30.28 (38.4%)	78.91 (100.0%)	3.09 (4.1%)	36.09 (47.4%)	11.76 (15.5%)	25.14 (33.0%)	76.08 (100.0%)
Gold	3.30 (4.2%)	34.05 (43.5%)	10.17 (13.0%)	30.68 (39.2%)	78.20 (100.0%)	20.28 (19.3%)	61.99 (59.0%)	7.33 (7.0%)	15.54 (14.8%)	105.14 (100.0%)
Platinum (regional committee)	1.19 (1.5%)	37.87 (48.9%)	8.68 (11.2%)	29.72 (38.4%)	77.46 (100.0%)	0.00 (0.0%)	35.42 (56.6%)	2.84 (4.5%)	24.31 (38.9%)	62.57 (100.0%)
Platinum (national committee)	0.00 (0.0%)	32.92 (39.6%)	15.93 (19.2%)	34.26 (41.2%)	83.11 (100.0%)					

ACCEA, Advisory Committee on Clinical Excellence Awards.

In a sensitivity analysis excluding the NRES subcommittee score, we found only minimal changes to the variance component estimates and so they are not shown. It is also of note that the variance of mean application scores observed within the NRES subcommittee was similar to that observed in the other subcommittees.

Table 6 shows the reliability achieved with a varying number of assessors in the current situation where the assessors are kept the same for all applications at a given level within a particular committee (as judgements are made within committee, in this scenario the assessor variance and subcommittee variance are not considered important). The number of assessors required to reach various reliability thresholds is shown in table 7, where it is evident that the assessment of new bronze applications could attain a reliability of 0.9 with 12 assessors per committee; other levels would only reach around 0.8 with similar numbers of assessors.

**Table 6:** Reliability achieved using differing numbers of assessors in the current situation where the make-up of committee is constant for all assessments of one type within a subcommittee, by level and application type

Number of assessors	Reliability								
	New applications					Renewals			
	Bronze	Silver	Gold	Platinum (regional subcommittee	Platinum (national committee)	Bronze	Silver	Gold	Platinum (regional subcommittee)
1	0.44	0.32	0.25	0.23	0.32	0.34	0.32	0.32	0.10
2	0.61	0.49	0.40	0.37	0.48	0.51	0.48	0.49	0.19
3	0.70	0.59	0.50	0.47	0.58	0.61	0.58	0.59	0.26
4	0.76	0.65	0.57	0.54	0.65	0.67	0.65	0.65	0.32
5	0.80	0.70	0.62	0.59	0.70	0.72	0.70	0.70	0.37
6	0.82	0.74	0.67	0.64	0.74	0.76	0.74	0.74	0.41
7	0.84	0.77	0.70	0.67	0.76	0.78	0.77	0.77	0.45
8	0.86	0.79	0.73	0.70	0.79	0.81	0.79	0.79	0.48
9	0.87	0.81	0.75	0.72	0.81	0.82	0.81	0.81	0.51
10	0.89	0.83	0.77	0.74	0.82	0.84	0.82	0.83	0.54
11	0.90	0.84	0.78	0.76	0.84	0.85	0.84	0.84	0.56
12	0.90	0.85	0.80	0.78	0.85	0.86	0.85	0.85	0.58

**Table 7:** Numbers of assessors required to reach various reliability thresholds in the current situation where the make-up of committee remains static for all assessments of one type within a subcommittee, by level and application type

	New applications					Renewals			
Reliability	Bronze	Silver	Gold	Platinum (regional subcommittee)	Platinum (national committee)	Bronze	Silver	Gold	Platinum (regional subcommittee)
0.7	4	5	8	8	6	5	5	4	20
0.8	6	9	13	14	9	8	9	9	35
0.9	12	20	28	31	20	18	20	20	78

Varying the make-up of groups of assessors such that they are not consistent for candidates being judged against one another substantially increases the number of assessors needed to reach the same levels of reliability compared to the current situation (see online supplementary material). While there was some evidence that assessors from different background roles gave systematically different scores, adjusting for this had no appreciable impact on reliability (see online supplementary material).

## Discussion

We have outlined, for the first time, the evidence-base in respect of the assessment process applied to applications by senior clinicians for a clinical excellence award. These awards are prestigious, and highly competed for, with only around 15% of NHS consultants or senior academic general practitioners holding such an award during their lifetime. The awards themselves are of significant monetary value, estimated to total £160 m (US$230 m) across the NHS in 2015–2016, and it is of importance that the assessment process should be transparent and reliable.

We explored the ACCEA awards process operated in the 2014 round of applications. Nearly 2000 applications were submitted. In each centre, between 6 and 12 assessors were used to make an overall judgement about the suitability of each award. The same scoring scale was used across each award level. The validity of the assessment process was therefore suggested by a predictable increase in scores across the four award grades, evident for new and renewal applications.

This study has identified that reliable assessments (>0.7) of bronze applications can be attained by using just 4 assessors, in contrast with the average of around 10 assessors used across the whole sample. Reliability can be increased by increasing the number of assessors; for example, in assessing bronze-level applications, reliability of 0.8 and 0.9 can be attained through the use of 6 and 12 assessors, respectively. The decision regarding the appropriate level of reliability to adopt is ultimately pragmatic, being based on the number of available assessors, given the present structures and resources within which the assessment process operates. A threshold of 0.9 for reliability may be judged appropriate for some ‘high stakes’ assessments such as in final medical examinations.^8^ While it might be argued that a somewhat lower threshold for reliability would also be defensible in settings such as we describe here, given the nature and purpose of the awards, we would advise that a reliability threshold of at least 0.8 be adopted where possible.

### Strengths and limitations

The main strength of the study is that it used comprehensive national data drawn from all of the 16 subcommittees assessing applications in the UK. Furthermore, we have used a modelling approach that quantifies variances from a number of sources. A limitation of our analysis is that we assume the assessor variance to be constant across different types of assessor. We are unable to determine empirically if this assumption is reasonable owing to a lack of statistical power, but we have no reason to suspect that it is an unreasonable assumption. We also assume that the assessor variances are constant across committees, and while there is some evidence that a committee made up of more experienced assessors are more consistent, this is unlikely to have a large impact on our findings as regional committees all contain a range of experience in their assessors.

It is, perhaps, not surprising that reliability is harder to achieve among higher level award applicants and among applicants for renewal of awards. This is primarily because such individuals tend to be more ‘similar’ in profile, harder to differentiate and thus require larger numbers of assessors. Thus, achieving reliability >0.8 in the assessment of new gold or platinum applications requires 13 and 14 assessors, respectively, although reliability of this level can be attained in scoring by 9 assessors in a national platinum subcommittee (the national platinum subcommittee currently involves around 28 assessors, well in excess of what is actually required). Members of this latter subcommittee are drawn from around 28 experienced, senior members of all of the subcommittees, and their experience may result in more consistent scoring, in turn accounting for the greater reliability observed following scoring by these assessors. At its most extreme, scoring by 78 experienced individuals is required to attain reliability >0.9 in the assessment of platinum renewal applications—an assessor quotient unlikely to be achievable within the present scoring structures.

An alternative approach to improving reliability is to consider that the scoring system may be insufficiently sensitive to detect differences between applicants, especially differences among those applying for higher level awards. Taking the example of platinum applications, for which the vast majority of scores are either ‘excellent’ contributions (scoring 10) and ‘over and above contractual requirement’ (scoring 6), the scoring system allows little room for differentiation between the very best applicants. It is interesting to note that the national committee do seem to use the instrument in a more discriminating way, but potentially still not good enough to attain a reliability threshold of 0.9. In these circumstances, one option to consider would be the design of a more sensitive instrument, perhaps involving the more accurate definition of performance at each of the scoring threshold, or perhaps offering more scoring options to allow greater discrimination. In addition, and importantly, training of all assessors is likely to result in improved overall reliability of the assessment process.

Despite being resourced by the most experienced assessors, we observed that assessments undertaken by NRES had similar variance to that seen in other subcommittees. Theoretically, one would anticipate that the additional experience of assessors would result in greater reliability of assessments. On reflection however, our observation is not surprising, since applications considered by NRES are likely to be rather similar to other applications made at the same level being considered by NRES, since all may be considered ‘borderline’—this being the basis for referrals to NRES. The greater potential reliability offered by experience of assessors thus appears to be offset by the greater similarity among those being assessed, accounting for the observed similarity in variance.

In the current situation, where all assessors score all applicants for a given level within a subcommittee, the make-up is standard for all applicants within that assessor scoring group. Owing to this, there is no gain to be made by standardising committee make-up in terms of assessor background role. In theory, it may help in the situation where comparisons are made between applicants scored by different assessors (such as using a national threshold for award), but the improvements are negligible compared to the dramatic reduction in reliability this reconfiguration would incur. Given the observed patterns of scoring and reliability, consideration should be given to splitting the subcommittee unequally with a view to ensuring that larger numbers of assessors are allocated to score applications above the bronze level. Thus, a subcommittee of 22 people might allocate 8 assessors to the assessment of bronze-level applications, and 14 individuals to the remaining applications.

## Conclusions

Assessment processes pertaining in the competitive allocation of public funds need to be credible and efficient. The present arrangements for assessing and scoring applications are defensible, depending on the level of reliability judged to be required in the assessment process. Our data suggest that some relatively minor reconfiguration in approaches to scoring might usefully be considered to further refine and optimise the use of available resources deployed in the assessment of these prestigious awards.
