# Supplementary material for: Clinical excellence: evidence on the assessment of senior doctors' applications to the UK Advisory Committee on Clinical Excellence Awards. Analysis of complete national data set
Source: BMJ Open. 2016 Jun 2;6(6):e011958. doi: 10.1136/bmjopen-2016-011958 (PMC4893866; doi:10.1136/bmjopen-2016-011958)
Supplement: Supplementary data [file bmjopen-2016-011958supp.pdf]

Clinical excellence – evidence on the assessment of senior doctor's applications to the UK Advisory Committee on Clinical Excellence Awards

Online Supplementary Material

Judgement about awards are made by sub-committees which apply specific regional cut-offs to decide when awards are recommended (rather than applying national cut-offs). Because of this for the main analysis we estimated reliabilities based on scores derived within sub-committee rather than as a part of a national spectrum. In this situation, where all applicants at a given level are assessed by all of the same assessors, any variation in the hawkish/dovish tendency of individual assessors will not affect reliability, as the same exaggeration/suppression of scores will apply equally to all candidates within a sub-committee. Thus the assessor variance does not influence reliability (λ) which is given by:

$$\lambda = \frac{\sigma_{AP}^2}{\sigma_{AP}^2 + \frac{\sigma_\varepsilon^2}{n}} \qquad 1$$

where n is the number of assessments made per candidate, σ_{AP}^2 is variance attributable to applicants, and σ_ε^2 is error variance.

In the contrasting situation, for example where the make-up of groups of assessors is not consistent for candidates being judged against one another the assessor variance must be taken into account. Situations where this might apply include ones where groups of assessors did not all see all of the applications being assessed or a national threshold score for an award was set rather than a local one. In such situations the reliability is then given by a different equation:

$$\lambda = \frac{\sigma_{AP}^2}{\sigma_{AP}^2 + \frac{\sigma_{AS}^2 + \sigma_e^2}{n}} \qquad 2$$

which incorporates σ_{AS}^2, variance attributable to assessors.

Supplementary Table 1 shows how the number of assessors required increases if the committee make up changed between applications. Here the required number of assessors becomes much higher than those required in the current situation and acceptable levels of reliability are unlikely to be reached for all but new bronze applications.

Finally, when we tested whether assessors from different background roles gave systematically different scores we only found evidence that this was the case for new bronze applications ($p=0.001$, $p>0.1$ in all other cases). This may in part reflect a lack of power due to the smaller sample sizes for higher awards. For new bronze applications (where we had the highest available sample size, and so best available power) lay assessors gave, on average the highest scores (4.7 points higher than employer assessors, 95%CI 2.0, 7.3) with clinical assessors not discernibly different from employer assessors (2.0 points higher than employer assessors, 95%CI -0.4, 4.5). However, taking account of observed differences (regardless of the test statistic) explained only a very small proportion of the observed variance attributable to assessors, and hence made minimal difference to the number of assesors presented in Supplementary Table 1 and hence are not shown.

Reliability	New Applications					Renewals			
	Bronze	Silver	Gold	Platinum (Regional sub-committee)	Platinum (National committee)	Bronze	Silver	Gold	Platinum (Regional sub-committee)
0.7	6	11	15	19	10	10	13	25	50
0.8	11	18	26	32	17	17	21	43	85
0.9	23	40	58	71	38	37	47	96	190

Supplementary Table 1 –Numbers of assessors required to reach various reliability thresholds in the alternative situation where the makeup of committee changes between assessments of one type within a sub-committee, by level and application type.
